# Halo fluorescence fibrinolysis test: a novel quantitative assay to evaluate fibrinolysis on established plasma clots

**DOI:** 10.1016/j.rpth.2025.102874

**Published:** 2025-04-27

**Authors:** Zikou Liu, Orr Zaacks, Be’eri Niego, Robert L. Medcalf

**Affiliations:** 1 Molecular Neurotrauma and Haemostasis Laboratory, Australian Centre of Blood Diseases, School of Translational Medicine, Monash University, Melbourne, Victoria, Australia; 2 NanoBiotechnology Laboratory, Australian Centre for Blood Diseases, School of Translational Medicine, Monash University, Melbourne, Victoria, Australia

**Keywords:** fibrinolysis, fluorescence, plasma, tissue-type plasminogen activator, thrombolytic therapy

## Abstract

**Background:**

Fibrinolysis is essential for dissolving blood clots and maintaining hemostasis. This process is primarily mediated by tissue-type plasminogen activator, which converts plasminogen into plasmin, thereby breaking down fibrin clots. Traditional amidolytic assays often measure plasmin generation without directly assessing fibrin degradation, while thromboelastography frequently overlooks clot maturation, which significantly influences fibrinolysis resistance.

**Objectives:**

To address these limitations, we present a novel quantitative assay for analyzing fibrinolysis on established clots, termed the halo fluorescence fibrinolysis (HoFF) test.

**Methods:**

The HoFF test used fluorophore-conjugated fibrinogen to form halo-shaped plasma clots. Fibrinolysis was induced with tissue-type plasminogen activator or its variant tenecteplase, and clot breakdown was monitored via real-time fluorescence detection by a microplate reader. The fluorescence signal was analyzed to calculate a fibrinolysis index, indicating fibrinolytic capacity. Its specificity for fibrinolysis over plasmin generation was validated against traditional amidolytic assays using a plasmin substrate.

**Results:**

Fluorescence-labeled fibrinogen was confirmed as a reliable marker of fibrin degradation. The HoFF test exhibited strong linear correlations between the fibrinolysis index and plasminogen activator concentrations, with robust reproducibility. It also effectively evaluated tenecteplase-induced fibrinolysis and demonstrated versatility across clot types, including mouse plasma and human whole-blood models. Furthermore, the test distinguished fibrinolysis from plasmin generation, demonstrated by the differential effects of tranexamic acid inhibition.

**Conclusion:**

The HoFF test offers a sensitive, reliable, and high-throughput tool for quantitatively evaluating fibrinolysis on established human and mouse plasma and whole blood clots.

## Essentials

• The need to assess fibrinolysis directly on established clots remains unmet in the field.
• The HoFF test uses labeled fibrinogen to quantitatively demonstrate fibrinolysis in real time.
• The HoFF test assesses fibrinolysis in clots formed in human and mouse plasma and whole blood.
• This high-throughput assay provides a sensitive, cost-effective tool for fibrinolysis research.

## 1 Introduction

Fibrinolysis is a physiological mechanism that orchestrates the dissolution of thrombi, thereby counterbalancing coagulation and maintaining hemostasis [1,2]. The process of fibrinolysis is mediated by the action of tissue-type plasminogen activator (tPA) on the circulating zymogen, plasminogen, to create the potent serine protease, plasmin. Both tPA and plasminogen bind to the lysine residues on the fibrin clot surface, facilitating localized plasmin generation and significantly accelerating fibrinolysis [3]. Indeed, tPA-mediated plasminogen activation is vastly enhanced by fibrin, which acts as an essential cofactor for the entire process of clot removal.

Recombinant tPA (alteplase) and its genetically modified variant tenecteplase (TNK), with a longer plasma half-life, have been the frontline pharmacologic interventions for acute ischemic stroke and myocardial infarction to induce thrombolysis and blood reperfusion [4]. On the other hand, tranexamic acid (TXA), which can competitively inhibit plasminogen binding to fibrin surfaces and suppress fibrinolysis, is widely used in various clinical scenarios to prevent excessive bleeding [[5], [6], [7]]. While TXA is generally effective and safe as an antifibrinolytic agent, blood clot removal by tPA or TNK is not always efficacious and may even induce serious adverse complications [8,9]. Hence, the assessment of drug impact on fibrinolysis plays a crucial role in guiding treatment decisions.

Over the years, numerous tests have been developed to assess fibrinolytic capacity, yet a universally accepted quantitative standard remains undefined [10,11]. Amidolytic assays employing chromogenic plasmin substrate (ie, S-2251) provide a means of determining plasmin generation induced by plasminogen activators, yet the process of fibrinolysis itself is not included in the assessment [12]. In fact, plasmin generation does not necessarily imply fibrin degradation [13]. Viscoelastic tests (such as thromboelastography) and clot lysis assays are utilized to determine the impact of fibrinolytic agents. However, it needs to be highlighted that therapeutic fibrinolysis (thrombolysis) is undertaken to remove established blood clots, and this is not accurately reflected in viscoelastic or clot lysis assays when the fibrinolytic agents are added at the onset of clotting process [14,15]. Since clot structure and its mechanical properties, such as fiber thickness and surface rigidity derived from clot maturation, significantly influence resistance against fibrinolysis [16], a test based on established clots is more desirable for translational studies.

In this study, we developed a novel quantitative assay for analyzing fibrinolysis on established clots, termed “halo fluorescence fibrinolysis (HoFF) test.” This method builds on clots formed in a halo shape around the perimeter of a well in a 96-well plate, leaving the optical compatible center area ideal for light-based detection [17,18]. Our method harnesses fluorescence-labeled fibrin(-ogen) as a tracer to represent clot degradation. Through analyzing the real-time recorded fluorescence signal by a microplate reader, we generated a fibrinolysis index (FI) that linearly correlates with the concentration of plasminogen activators, reliably and quantitatively revealing the fibrinolytic capacity. The HoFF test demonstrates potential adaptability for evaluating fibrinolysis in clots derived from mouse plasma, human whole blood, and hypofibrinolytic human plasma. Furthermore, we validated the assay’s specificity in distinguishing fibrinolysis from plasmin generation by comparison with the S-2251 amidolytic assay.

## 2 Methods

### 2.1 HoFF test

**Figure 1:**
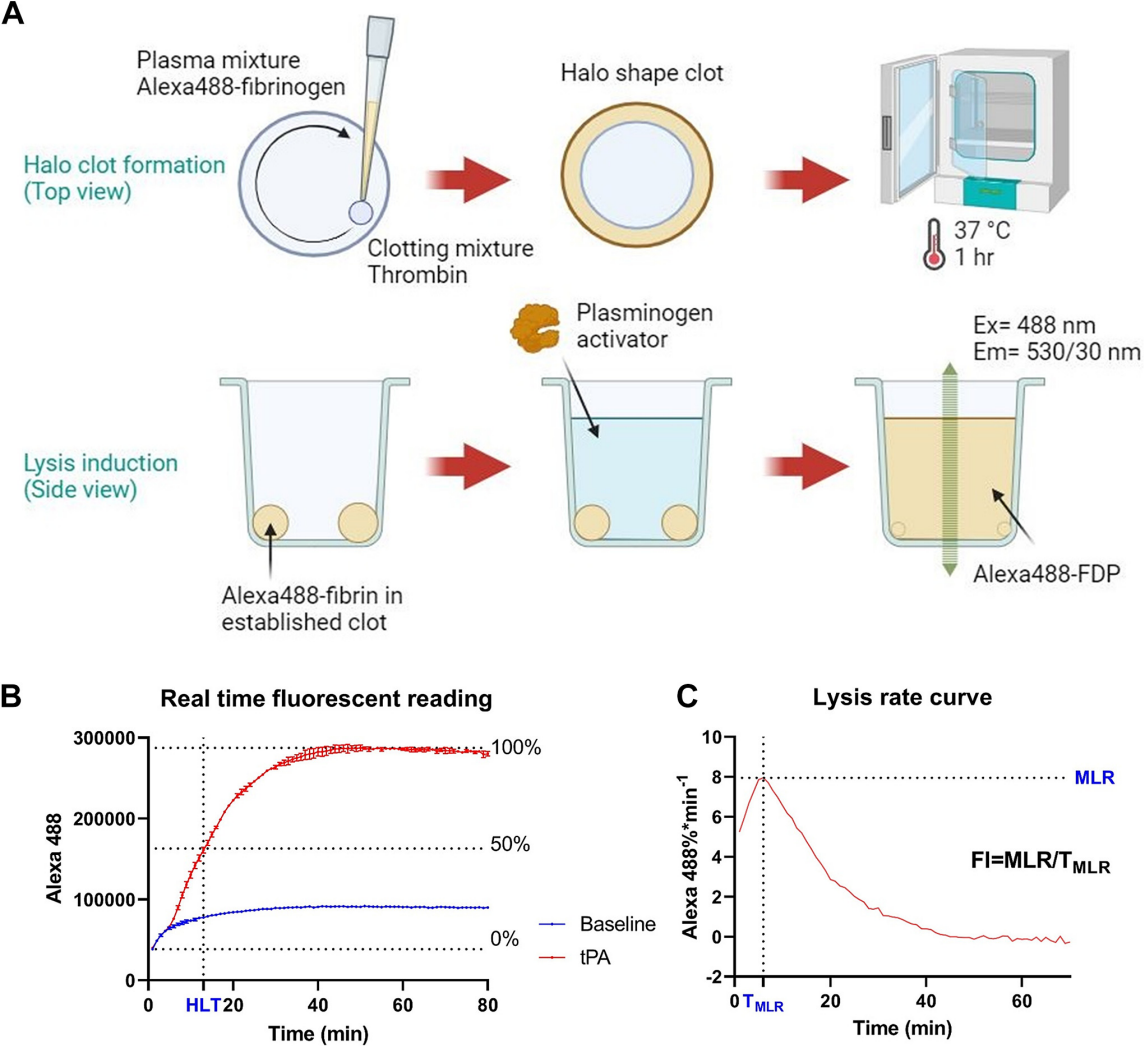
Halo fluorescence fibrinolysis test procedure and data analysis. (A) two stages of the halo fluorescence fibrinolysis test: halo plasma clotting (top view) by mixing the plasma containing Alexa Fluor 488-conjugated purified human fibrinogen (Alexa488-fibrinogen) with thrombin, followed by 1-hour incubation, and lysis induction (side view) by adding plasminogen activator on top of the halo clot. Graphs were made with Biorender.com. (B) real-time fluorescence readings tracking fibrinolysis, comparing baseline with 15 nM tissue-type plasminogen activator (tPA) treatment, indicating the half-lysis time (HLT). (C) processed data from (B) showing the lysis rate curve by percentage, with maximum lysis rate (MLR) and the time of MLR (T_MLR_) marked, and the formulation of fibrinolysis index (FI) included. Em, emission; Ex, excitation; FDP, fibrin degradation product.

This method includes the formation of a fluorophore-containing plasma clot in halo shape and the following clot lysis induced by thrombolytics added on top of the clot. The process is illustrated in Figure 1A.

#### 2.1.1 Halo plasma clot formation

The optimal concentration of Alexa Fluor 488-conjugated purified human fibrinogen (F13191, Thermo Fisher Scientific, abbreviated as Alexa488-fibrinogen) for signal detection by a microplate reader (CLARIOstar Plus, BMG Labtech) was determined to be 1.5 μg/mL in a total volume of 80 μL in a well of 96-well flat-bottom transparent plate (Corning Costar 96-Well, Cell Culture-Treated, and Flat-Bottom Microplate). Thrombin (24 U/mL; Bovine, #605157, Millipore) was prepared in solution of 400 mM CaCl_2_ and 150 mM NaCl, termed “clotting mixture.” Platelet-poor plasma collected from healthy human donors (approved by Monash University Human Research Ethics Committee [project ID: 35867]) or wild-type C57bl/6 mice (animal ethics approval E/2007/2020/M) was diluted 1:1 with 150 mM NaCl solution. In this plasma dilution, Alexa488-fibrinogen was added at 4.8 μg/mL to reach final concentration of 1.5 μg/mL when adding thrombolytics on top. This mixture was termed “plasma mixture.” When mentioned, TXA (gifted from recently completed clinical trial “Prehospital Antifibrinolytics for Traumatic Coagulopathy and Hemorrhage” [19]) was added to the plasma mixture at the indicated dosage. When mentioned, plasminogen activator inhibitor-1 (PAI-1; #A8111-25UG, Sigma–Aldrich) was added to the plasma mixture at the indicated dosage to induce hypofibrinolytic condition in plasma clots.

To form a halo-shaped clot, a 5 μL droplet of the clotting mixture was pipetted at the bottom corner of testing wells in the 96-well plate. The plasma mixture (25 μL) was added to the 5 μL clotting mixture (final thrombin concentration used was 4 U/mL). A halo-shaped clot was made following method described by Bonnard et al. [17]. Duplicate wells were included for each testing condition. The plate was then incubated at 37 °C for 1 hour to obtain established plasma clots.

#### 2.1.2 Induction of fibrinolysis and fluorescent signal detection

Alteplase (tPA) or TNK (both from Boehringer Ingelheim) dialyzed against 0.4 M HEPES, pH = 7.4, was prepared in 150 mM NaCl solution at indicated concentrations, termed “reaction mixture” and preloaded in wells adjacent to the clots. The reaction mixture (50 μL) was added on top of the clots. The detection of fluorescence signal (excitation [Ex] = 488 nm, emission [Em] = 530/30 nm) by a microplate reader was initiated immediately without shaking. The plate reader was set to plate mode (slow kinetics), scanning every well in the center area at 1-minute intervals (10 flashes per read) over a period of 60 to 90 minutes. The average readings from duplicate wells were generated to present the outcome.

#### 2.1.3 Data processing and analysis

Each testing plate included non-lysis control wells (reaction mixture was replaced with 150 mM NaCl solution) and full-lysis control wells (both clotting mixture and reaction mixture were replaced with 150 mM NaCl solution). From these wells, the initial fluorescence readings were used to represent 0% lysis $\left({Alexa488}_{0\%}\right)$ and 100% lysis $\left({Alexa488}_{100\%}\right)$, respectively. The real-time recorded fluorescence readings from sample wells were first normalized by the fluorescence increase from 0% lysis to 100% lysis $\left({Lysis}_{\%}=\frac{Alexa488-{Alexa488}_{0\%}}{{Alexa488}_{100\%}-{Alexa488}_{0\%}}\times 100\%\right)$. The half-lysis time (HLT) was determined as the time (minutes) to reach 50% lysis for each recording (Figure 1B). The first derivative of the lysis percentages in time (minutes) was calculated and then smoothed by a 10-point moving average, which represented the lysis rate $\left(LysisRate=\frac{\Delta {Lysis}_{\%}}{\Delta t}\right)$. To remove the background signal, the baseline signal from the non-lysis control wells was subtracted from each testing sample at each time point (Figure 1C). The peak of this adjusted lysis rate curve was termed “maximum lysis rate” (MLR), and the corresponding time point was referred to as the time of MLR (T_MLR_). The ratio of MLR and T_MLR_ was termed Fibrinolysis Index, “FI” $\left(FI=\frac{MLR}{T_{MLR}}\right)$.

For the HoFF test using whole blood instead of plasma to make the clots, the fluorophore was replaced with Alexa Fluor 647-conjugated purified human fibrinogen (F35200, Thermo Fisher Scientific, abbreviated as Alexa647-fibrinogen) at the same concentration. Fluorescence detection parameters were set to Ex at 640 nm and Em at 680/10 nm.

### 2.2 S-2251 amidolytic assay

The procedure was modified from a previously published method for plasma-based amidolytic assays [18]. Similar to HoFF test, the process of this amidolytic assay also includes formation of a halo-shaped plasma clot as the first step; however, the plasma mixture was prepared without the Alexa488-fibrinogen. A plasmin-specific chromogenic substrate (S-2251, Chromogenix) was added to the reaction mixture (containing thrombolytics) at 1.43 mM to reach a final concentration of 1 mM (volume = 100 μL). The reaction mixture (70 μL) was added to the established plasma clots to induce the activation of plasminogen in the clot. The cleavage of S-2251 by plasmin generates p-nitroaniline that can be detected by a microplate reader by reading the light absorbance at 405 nm wavelength. The signal-acquiring program was set in the same manner as in the HoFF test. The first derivative of the light absorbance ($R=\frac{\Delta Abs405}{\Delta t}$) was generated and smoothed by a 10-point moving average to indicate the substrate cleavage rate, which corresponds to the real-time plasmin catalytic activity and concentration. The tPA-induced plasmin generation rate (PGR) was determined as the slope of the rate curve from the starting point to the maximum point $\left(PGR=\frac{R_{\max}-R_{0}}{T_{\max}}\right)$.

### 2.3 Gel electrophoresis and Western blot

Samples were collected in sample loading buffer (1% sodium dodecyl sulfate, 10% glycerol, 100 mM Tris-HCl, 0.5% bromophenol blue dye), centrifugated at 13,000 × *g* for 2 minutes, and the soluble fractions were resolved under nonreducing conditions by sodium dodecyl sulfate–polyacrylamide gel electrophoresis (4%-15% Mini-Protean, precast protein gel, Bio-Rad). For Western blot, proteins were transferred onto polyvinylidene difluoride membranes and hybridized with antibodies against human fibrinogen (ASHFBGN-GF-HT, rabbit, Molecular Innovation), human plasminogen (SASHPLG-GF-HT, sheep, Molecular Innovation), and human D-dimer (MCA25623, mouse, Bio-Rad). Both Alexa488-fibrinogen fluorescent signal directly on the gel and the chemiluminescent signal on the blot membrane were obtained by ChemiDoc Imaging System (Bio-Rad).

## 3 Results

### 3.1 Validation of fluorescence-labeled fibrinogen-derived components as a marker of fibrinolysis

Alexa488-fibrinogen was added to the plasma mixture in the HoFF test as a marker to represent the conversion of endogenous fibrinogen to fibrin in the clotting phase and fibrin degradation products (FDPs) in the following lysis phase. To validate this representation, we collected samples at indicated time points during the HoFF test by adding sample loading buffer (containing 1% sodium dodecyl sulfate) to different wells to stop the reactions. Protein components were subjected to sodium dodecyl sulfate–polyacrylamide gel electrophoresis.

**Figure 2:**
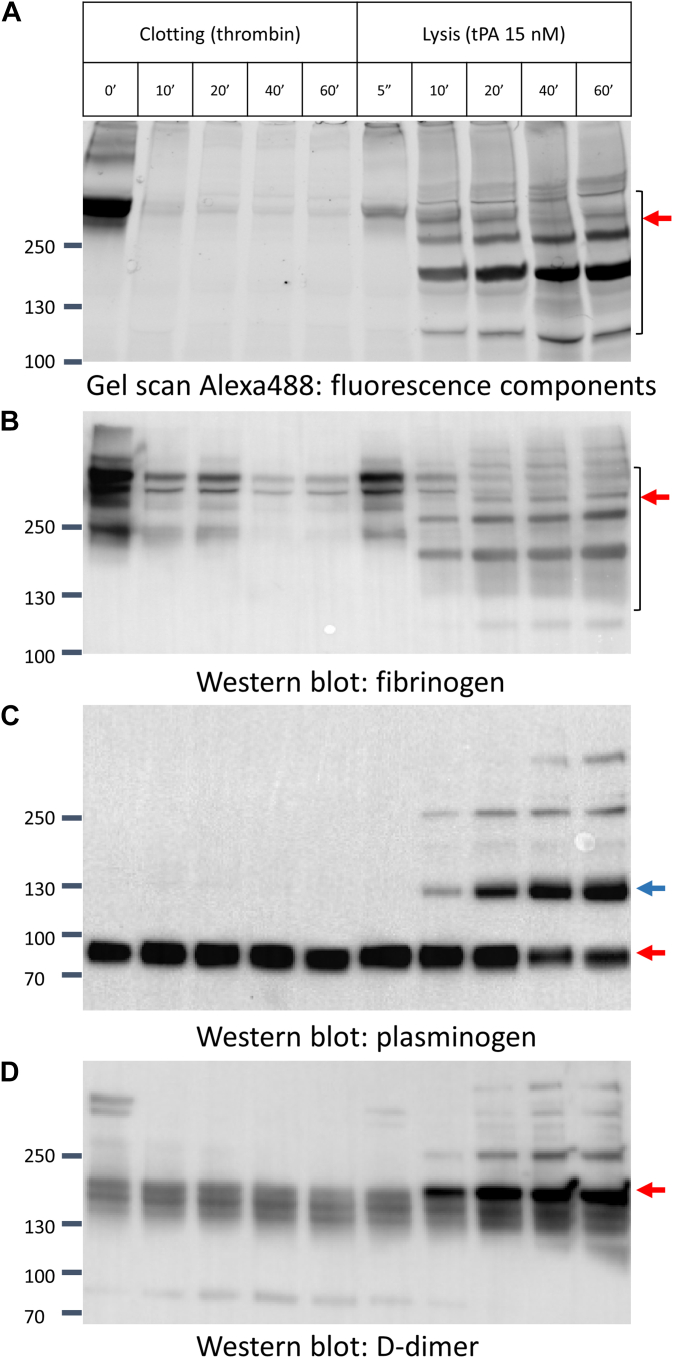
Fluorescent signal represents the process of fibrinolysis. Samples collected from the halo fluorescence fibrinolysis test at different time points were resolved by sodium dodecyl sulfate–polyacrylamide gel electrophoresis. (A) fluorescence signal was detected by gel scanning for Alexa Fluor 488-conjugated purified human fibrinogen (Alexa488) in ChemiDoc Imaging System to show the fibrinogen and its derived species. The arrow points to fibrin degradation products. (B) Western blot using multiclonal antibodies against fibrinogen detected fibrinogen and fibrin degradation products. (C) Western blot using antibodies against plasminogen detected free plasmin(ogen) (red arrow) and plasmin–antiplasmin complex (blue arrow) induced by tissue-type plasminogen activator (tPA). (D) Western blot using antibodies against D-dimer shows the accumulation of D-dimer during fibrinolysis.

As shown in the Alexa488-fibrinogen gel scan (Figure 2A), full-length labeled fibrinogen (340 kDa) collected from unclotted plasma was evident at the start (first lane at left). Following thrombin treatment and clot formation, the fibrinogen was converted into insoluble fibrin, with little fibrinogen remaining in the soluble fractions. However, after treatment with tPA, the fluorescent signal emerged again, first at 340 kDa and later accumulating at lower molecular weight components, representing the lysis of the clot and formation of FDPs. The Western blot (Figure 2B) using multiclonal antibodies against fibrinogen also detected fibrinogen and its derived components, showing similar pattern. Furthermore, blots against plasminogen showed increased formation of plasmin–antiplasmin complex, indicating the generation of plasmin, which was quickly captured by α2-antiplasmin, while the blot detecting D-dimer showed the accumulation of FDPs (Figure 2C and D), as expected. Therefore, these results demonstrated that the fluorescent signal can approximate the release of FDPs and can be used to monitor the process of fibrinolysis.

### 3.2 Quantification of induced fibrinolysis by HoFF test

**Figure 3:**
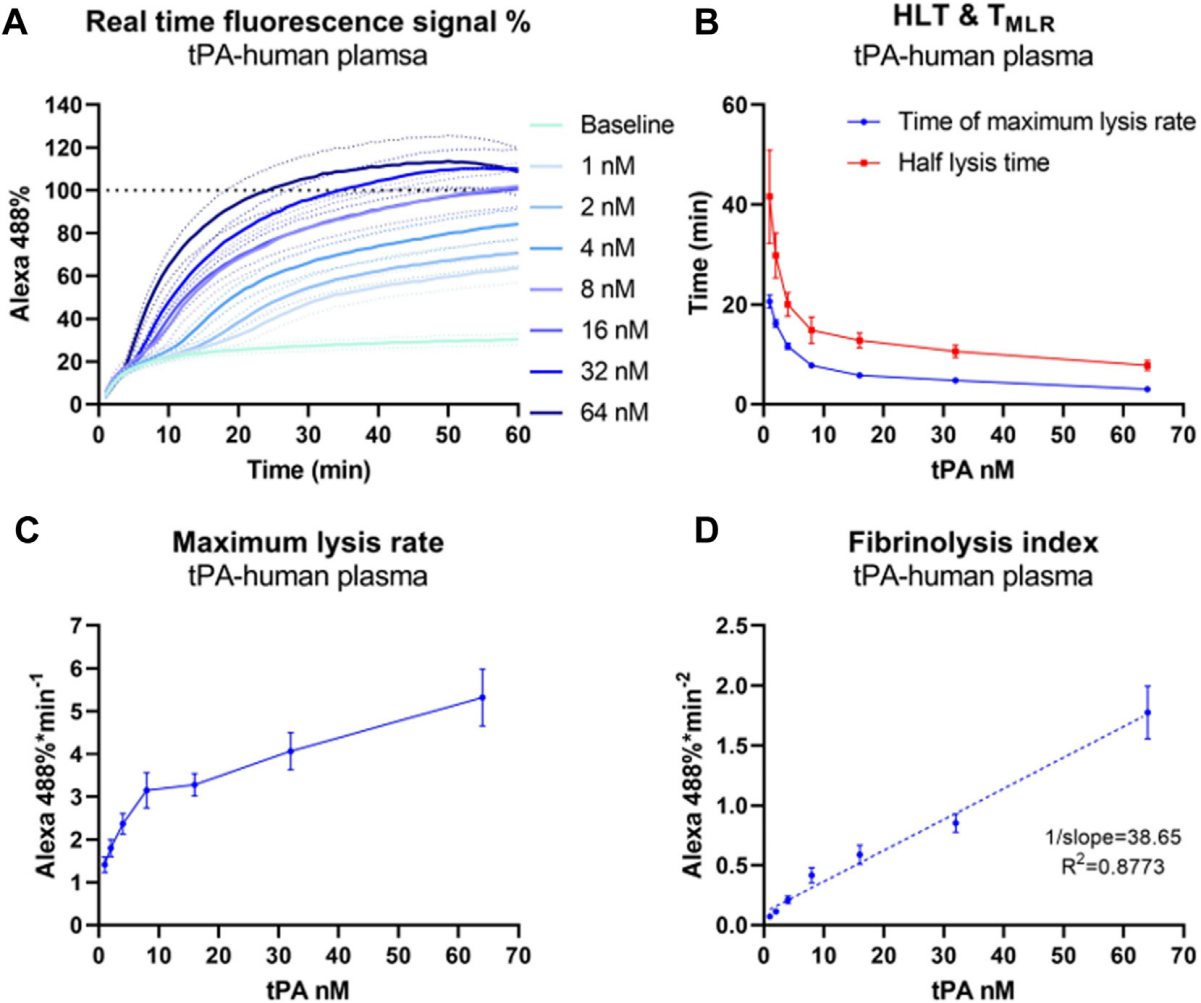
Quantification of tissue-type plasminogen activator (tPA)-induced fibrinolysis by the halo fluorescence fibrinolysis test. Varying concentrations of tPA (1-64 nM) were applied to lyse the human plasma clots in the halo fluorescence fibrinolysis test. (A) Alexa Fluor 488 (Alexa488) fluorescence signals were recorded over 60 minutes and normalized to the signal of a full lysis control. (B–C) indices of half-lysis time (HLT), time to maximum lysis rate (T_MLR_), and maximum lysis rate were plotted against the tPA concentrations. (D) fibrinolysis indices were determined and showed a linear correlation with tPA concentrations through regression analysis. Data are presented as the mean ± standard error of the mean (SEM). Plasma samples from 5 healthy donors were used for this experiment.

**Supplementary Figure 1:**
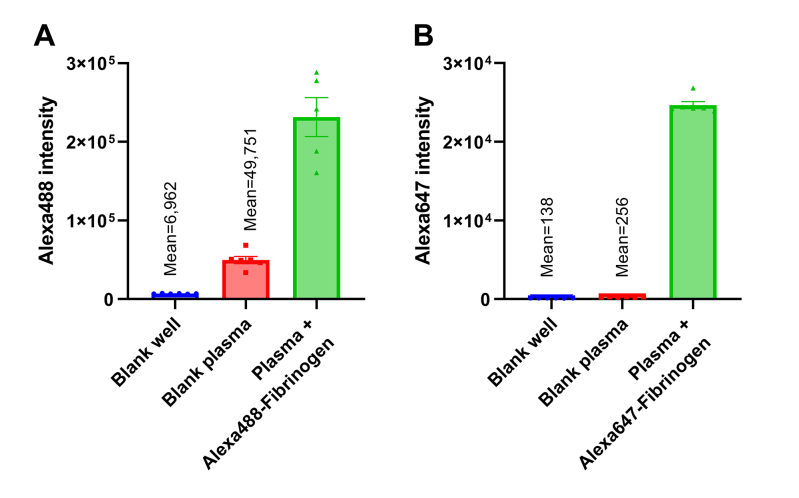
Differential autofluorescence signals from blank plasma detected in Alex488 and Alexa647 channels. Fluorescence intensity was measured in blank wells, blank plasma (without any fluorophore), and plasma containing fluorophore-conjugated fibrinogen (1.5 μg/mL). Fluorescence signals were detected using (A) Alexa488 channel (Ex=488 nm, Em=530/30 nm) and (B) Alexa647 channel (Ex=640 nm, Em=680/10 nm). Data are presented as mean ± standard error of the mean (SEM), with n ≥5. Em, emission; Ex, excitation

Having validated that the fluorescence signal was representative of fibrinolysis, we next aimed to determine the capacity of the HoFF test to quantitatively describe fibrinolysis induced by tPA, and to select the best index to represent the dose-dependent effects of tPA. A concentration gradient of tPA (1-64 nM) was first used to induce lysis in the HoFF test with human plasma clots. Alexa488-fibrinogen fluorescence signals were recorded and normalized to the signal of a full lysis control (Figure 3A). An initial increase in Alexa488-fibrinogen signal was observed under baseline conditions (no tPA was added). As shown in Supplementary Figure S1 (see later), this increase is due to the autofluorescence signal from soluble plasma component that diffused into the central area. This background signal was subtracted in following data analysis to reveal the tPA-specific impact (see Methods). Key indices were generated, including HLT, MLR, T_MLR_, and FI. Both HLT and T_MLR_ fit in log-decreasing curves as the tPA concentration increases, with T_MLR_ always preceding HLT at a given tPA concentration. Hence, the results can be obtained faster when using T_MLR_ for the assessment (Figure 3B).

On the other hand, MLR fits in a log-increasing curve with the tPA concentration; thus, these 3 parameters are all sensitive to tPA dosage at low dose range but plateau at higher tPA concentrations due to the consumption of plasminogen in the plasma clot (Figure 3C). MLR shows how dramatic lysis could occur at a specific time point, primarily depending on the concentration of accessible proteases and substrates, while T_MLR_ not only accounts for the kinetics of the lysis reaction but also considers delays caused by the physical properties of clots, such as surface rigidity and internal structure. We used the ratio of MLR to T_MLR_ to incorporate all these factors. This ratio is referred to as the FI. Using the same plasma sample, the FI had a strong linear correlation with tPA concentration (*R*^*2*^ = 0.8773; Figure 3D).

To confirm the reliability of the HoFF test, we used pooled human plasma from 10 healthy donors to make plasma clots instead of using plasma from different individuals in previous experiments. tPA at 4, 16, and 64 nM was applied to induce lysis, and FIs were generated for each reaction. The coefficients of variation (CVs) were calculated based on the SD and mean values (Mean) of FI $\left(\text{CV}=\frac{SD}{Mean}\times 100\%\right)$ . Two trials were included for each test, and 3 tests were performed at different times to generate the intraplate and interplate CVs, respectively, as shown in Table. The intraplate CVs were mostly below 10%, and the interplate CVs were <15%, indicating a robust consistency of the HoFF test.

**Table:** Coefficients of variation in the halo fluorescence fibrinolysis test.

FI	Plate 1				
	Trial 1	Trial 2	Mean	SD	CV
tPA 4 nM	0.172125	0.202157	0.187141	0.015016	8.023762
tPA 16 nM	0.587218	0.643192	0.615205	0.027987	4.549247
tPA 64 nM	1.920124	2.066698	1.993411	0.073287	3.676468

FI	Plate 2				
	Trial 1	Trial 2	Mean	SD	CV
tPA 4 nM	0.182482	0.17777	0.180126	0.002356	1.308049
tPA 16 nM	0.586453	0.546403	0.566428	0.020025	3.535236
tPA 64 nM	1.535541	2.247986	1.891763	0.356223	18.83019

FI	Plate 3				
	Trial 1	Trial 2	Mean	SD	CV
tPA 4 nM	0.172125	0.202157	0.187141	0.015016	8.023762
tPA 16 nM	0.587218	0.643192	0.615205	0.027987	4.549247
tPA 64 nM	1.920124	2.066698	1.993411	0.073287	3.676468

FI	Interplate (*n* = 6, *N* = 3)		
	Mean	SD	CV
tPA 4 nM	0.178393	0.017725	9.935709
tPA 16 nM	0.592221	0.057118	9.644796
tPA 64 nM	1.866568	0.247569	13.26331

Pooled plasma from 10 healthy donors was used to make the plasma halo clots in the halo fluorescence fibrinolysis test. tPA at 4, 16, and 64 nM was used to induce fibrinolysis. Two trials were included for each tPA dosage on each plate and the experiment was repeated on 3 plates at different times. Mean values and SDs were generated from the fibrinolysis indices in each trial. At each tPA dosage, the intraplate CVs are shown for each plate, and the interplate CVs were calculated from the 3 plates.

CV, coefficient of variation; FI, fibrinolysis index; tPA, tissue-type plasminogen activator.

### 3.3 Adaptations of the HoFF test for broader applications

The design of the HoFF test incorporates a halo-shaped clot, enabling light detection through the central area while measuring fluorescence signals indicative of fibrin-FDP release. This unique design presents significant potential for adaptation to other experimental models.

**Figure 4:**
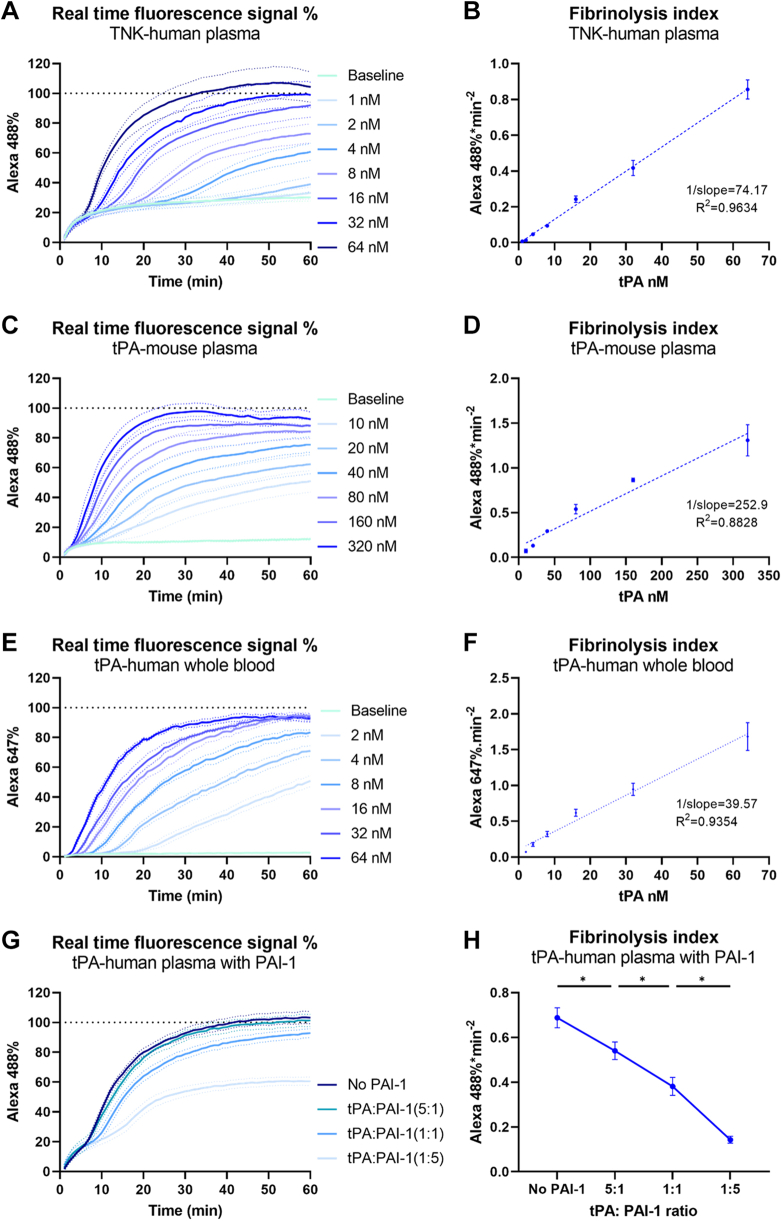
Adaptations of the halo fluorescence fibrinolysis test for broader applications. The halo fluorescence fibrinolysis test was used to assess fibrinolysis under various conditions: (A, B) tenecteplase (TNK)-induced fibrinolysis in human plasma clots (*n* = 5) over a concentration range of 1 to 64 nM, (C, D) tissue-type plasminogen activator (tPA)-induced fibrinolysis in mouse plasma clots (*n* = 4) over a concentration range of 10 to 320 nM, and (E, F) tPA-induced fibrinolysis in whole human blood clots (*n* = 3) over a concentration range of 2 to 64 nM. (G) plasminogen activator inhibitor-1 (PAI-1)-induced hypofibrinolytic conditions were evaluated in human plasma clots treated with 15 nM tPA (*n* = 3), and (H) fibrinolysis index showed a significant reduction with increasing PAI-1 levels. Data are presented as mean ± standard error of the mean (SEM). ∗Indicates a significant difference (*P* < 0.05). Alexa488, Alexa Fluor 488-fluorescence signal; Alexa647, Alexa Fluor 647-fluorescence signal.

We examined the fibrinolytic capacity of TNK on human plasma clots. Again, the real-time recording of fluorescence signal demonstrated the dosage effect of TNK (Figure 4A). By correlating concentrations with FI from the HoFF test (Figure 4B), the result showed a strong linear correlation (*R*^*2*^ = 0.9643). Based on the slopes from the linear regression, around double amount of TNK is required to induce same level of fibrinolysis in established plasma clots compared with tPA.

Building on the insights gained from the human plasma HoFF test, we extended its application to quantify fibrinolysis in mouse plasma clots. This approach, utilizing fluorescence signal detection, overcomes a significant challenge inherent in conventional global clot lysis assays, which rely on turbidity detection and are ineffective in mouse plasma due to its transparency, even when clotted. The HoFF test on mouse plasma clots revealed that human tPA effectively initiates fibrinolysis across a range of concentrations (Figure 4C). The FIs, reflective of tPA’s proteolytic capacity, also demonstrated a linear correlation with the concentration of tPA in mouse plasma (*R*^*2*^ = 0.8823; Figure 4D). However, based on the slopes of linear regression, inducing a comparable level of fibrinolysis in mouse plasma clots, as opposed to human plasma clots, required approximately 6-fold higher concentration of tPA, which corresponds to the relative resistance of mouse plasminogen to be activated by tPA [20].

**Supplementary Figure 2:**
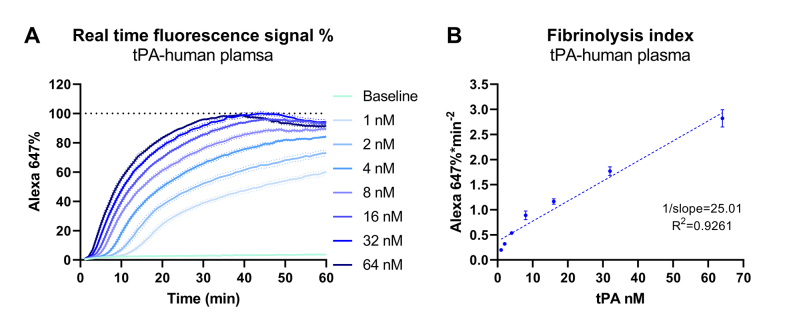
HoFF test using human plasma clots containing Alexa647-fibrinogen. (A) tPA (1-64 nM) was applied to lyse human plasma clots containing Alexa647-fibrinogen. Alexa647 fluorescence signals were recorded over 60 min and normalized to the signal of a full lysis control. (B) fibrinolysis Indices from (A) demonstrated a linear correlation with tPA concentrations. Data are presented as mean ± standard error of the mean (SEM), with n=6. tPA, tissue-type plasminogen activator

Whole blood differs from plasma as it contains blood cells and platelets, incorporating additional factors involved in coagulation and thrombolysis. Monitoring human whole blood clot lysis, therefore, provides a more physiologically relevant assessment of fibrinolysis in clinical scenarios. To adapt the HoFF test for whole blood clots, Alexa488-fibrinogen was replaced with Alexa647-fibrinogen, an infrared fluorophore, to circumvent interference from hemoglobin’s color. Fresh blood samples were collected from 3 healthy donors, and a range of tPA concentrations (2-64 nM) was tested to induce clot lysis. Notably, the Alexa647-fibrinogen signal exhibited very low background fluorescence when clots were treated with blank buffer (baseline; Figure 4E), minimizing the initial fluorescence increase observed in the HoFF test using Alexa488-fibrinogen. Furthermore, the FI correlated linearly with tPA concentration (*R*^*2*^ = 0.9354), demonstrating that the HoFF test can effectively evaluate fibrinolysis in whole blood clots (Figure 4F). As noted above, blank plasma (without any fluorophore) exhibited a high autofluorescence signal in the Alexa488-fibrinogen channel (Ex = 488 nm, Em = 530/30 nm) but a relatively low autofluorescence signal in the Alexa647-fibrinogen channel (Ex = 640 nm, Em = 680/10 nm) compared with blank wells (Supplementary Figure S1A, B). This prompted us to determine if Alexa647-fibrinogen could improve the sensitivity in the plasma clot-based HoFF test. As expected, the Alexa647-fibrinogen signal demonstrated a substantially reduced initial increase on baseline curve, which allowed earlier detection of deviating tPA curves compared with Alexa488-fibrinogen–based assay (Supplementary Figure S2A). Importantly, the linear correlation between FI and tPA concentration remained uninterrupted (*R*^*2*^ = 0.9261; Supplementary Figure S2B).

PAI-1 is the primary inhibitor regulating tPA activity within the circulatory system [21]. Consequently, the presence of PAI-1 within blood clots inhibits tPA-induced fibrinolysis. We aimed to assess how the hypofibrinolytic condition induced by PAI-1 could be detected using the HoFF test. To this end, varying concentrations of PAI-1 (6, 30, and 150 nM) were incorporated into a plasma mixture (25 μL for each clot) to form halo clots, followed by the addition of 15 nM tPA (50 μL for each clot) to induce fibrinolysis. The resultant molarity ratios of tPA to PAI-1 were 5:1, 1:1, and 1:5, respectively, with a no PAI-1 control group included as the baseline. The results demonstrated the Alexa488-fibrinogen signal was progressively attenuated with increasing PAI-1 concentrations (Figure 4G). The hypofibrinolytic condition was assessed using FI (Figure 4H). Statistical analysis confirmed a significant increase in fibrinolysis inhibition corresponding to higher PAI-1 levels.

### 3.4 Examining the capacity of the HoFF test to discriminate between fibrinolysis and plasmin generation

The release of fluorescence-labeled FDP directly reflects the breakdown of fibrin clots, which differs from plasmin generation. To examine whether the HoFF test can effectively discriminate between these 2 processes, we compared the blocking effect of TXA on fibrinolysis and plasmin generation measured by S-2251 amidolytic assays. TXA is an essential medication (antithrombolytic treatment) in many clinical scenarios for preventing excessive bleeding. As a lysine analog, TXA competitively binds to plasminogen, inhibiting plasminogen docking on the surface of fibrin clots, therefore suppressing fibrinolysis. However, TXA does not inhibit the ability of tPA to activate plasminogen in the absence of fibrin.

**Figure 5:**
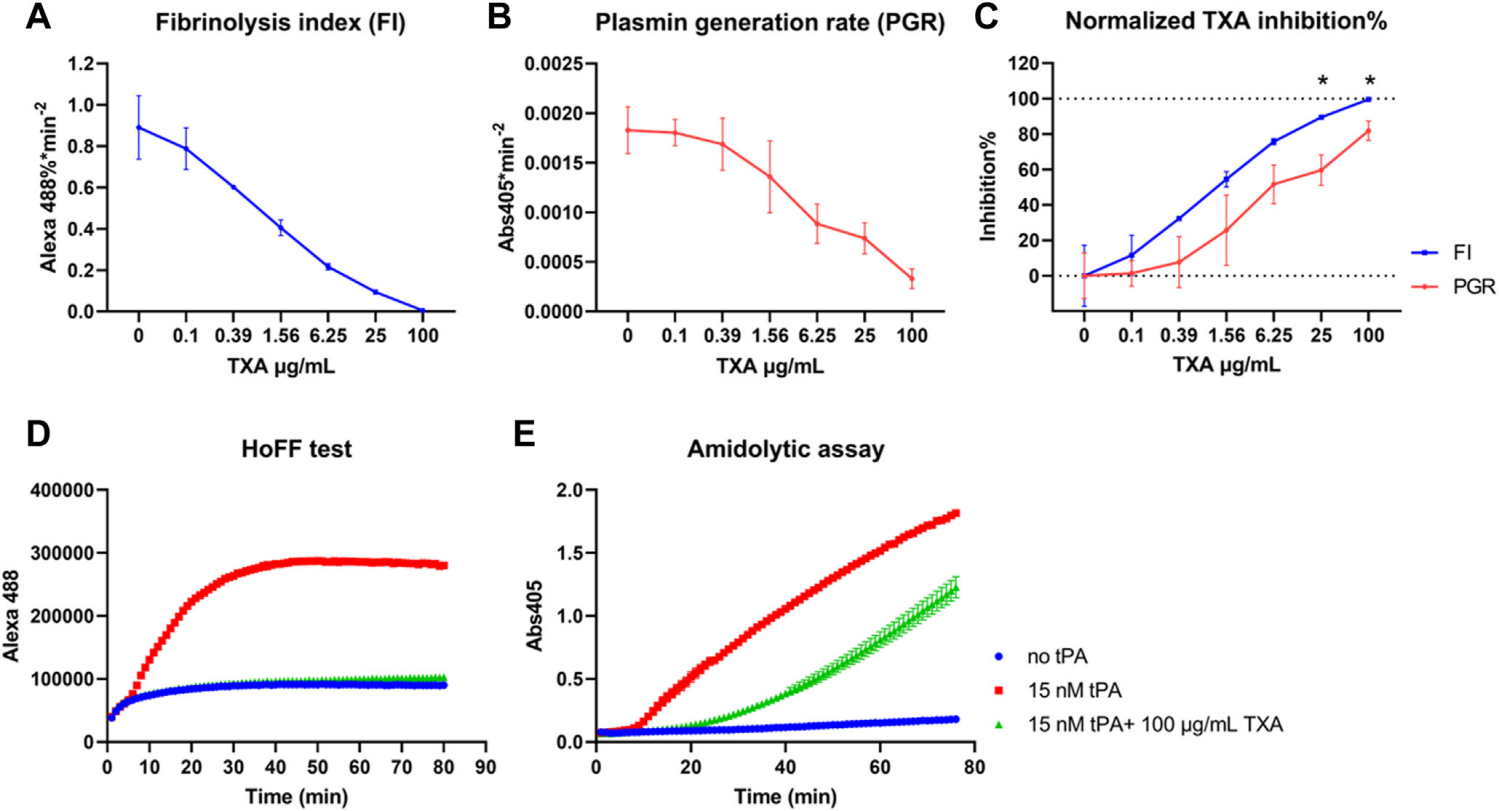
The halo fluorescence fibrinolysis (HoFF) test shows better sensitivity at detecting tranexamic acid (TXA) inhibition of fibrinolysis compared with the S-2251 amidolytic assay. The tissue-type plasminogen activator (tPA)-induced fibrinolysis was evaluated using HoFF test and Halo amidolytic assay, with an increasing dosage of TXA added to plasma mixture. (A–B) both fibrinolysis index (FI) from HoFF test and plasmin generation rate (PGR) from amidolytic assay showed inhibition of fibrinolysis by TXA. (C) TXA inhibition was normalized by taking non-tPA group as 0% fibrinolysis and non-TXA group as 100% lysis. The HoFF test showed an almost complete blockage of fibrinolysis by 100 μg/mL TXA, while the amidolytic assay showed that some plasmin could still be generated. Data are presented as mean ± standard error of the mean (SEM), *n* = 5, ∗*P* < 0.05. (D–E) representative plots of the raw readings from the HoFF test and S-2251 amidolytic assay showing the difference in fibrinolysis and plasmin generation with inhibition by 100 μg/mL TXA. The error bars (SEM) were generated from duplicate well readings within the same run. Abs405, absorbance at 405 nm; Alexa488, Alexa Fluor 488-fluorescence signal.

TXA (0.1-100 μg/mL) was premixed into the plasma halo clots, and fibrinolysis was induced by adding 15 nM tPA into the reaction. The FI from HoFF test was compared with the PGRs from the S-2251 amidolytic assay. Both FI (Figure 5A) and PGR (Figure 5B) showed a decline as TXA concentration increased. However, at the highest concentration of TXA, the FI was approaching zero, indicating a complete inhibition of fibrinolysis, yet plasminogen generation remained detectable. The difference was more obvious when normalizing the TXA inhibitory impact by taking “no TXA” group as “0% inhibition” (Figure 5C). The representative example showing the raw readings from the HoFF test and the S-2251 amidolytic assay clearly demonstrates the difference between the release of soluble FDP and the accumulation of cleaved plasmin substrate (Figure 5D and E). Hence, the S-2251 amidolytic assay evaluates plasmin formed both in the solution and on the fibrin surface, whereas the HoFF test is more specific to assess fibrinolysis *per se*.

## 4 Discussion

This study describes the development and evaluation of the HoFF test, a novel high-throughput quantitative assay to assess fibrinolysis on established clots.

We first validated the utility of fluorescence-labeled fibrinogen against endogenous fibrinogen in the plasma during clotting and fibrinolysis phases. As indicated by the manufacturer, each tracer fibrinogen molecule is conjugated with an average of 15 molecules of fluorescent dye, and therefore, the fluorescent signal would be present on a spectrum of fibrinogen-derived species. Supported by fluorescence scanning and Western blotting, the tracer was confirmed to accurately reflect the conversion of soluble fibrinogen to insoluble fibrin and subsequent release of soluble FDPs following treatment with tPA. The high sensitivity of fluorescence detection minimized the amount of tracer required. Less than 0.5% of labeled fibrinogen (4.8 μg/mL) mixed with endogenous fibrinogen (1 mg/mL) in the plasma mixture was sufficient to generate robust signal, which markedly reduces the likelihood of the tracer interfering with the natural clotting response. This also underscores its cost-effectiveness. In fact, when compared with the traditional S-2251 amidolytic assay, the HoFF test incurs <2% of the cost per testing well, making it a notably economical option for high-throughput screening tests.

The HoFF test introduces several analytical indices derived from real-time fluorescence readings, all sensitively depicting the gradient of tPA concentrations. Among them, FI offers a precise evaluation by mathematically modeling fibrinolysis as a second-order reaction from plasminogen activation. It demonstrated a tight linear correlation with plasminogen activator concentrations, effectively quantifying fibrinolytic capacity. In addition, we were able to extend the application of the HoFF test to not only assess the fibrinolytic strength of TNK on human plasma clots but also the capacity of tPA to lyse mouse plasma clots, human whole blood clots, and hypofibrinolytic human plasma clots containing PAI-1, demonstrating the potential of adaptation for broader applications.

A key advantage of the HoFF test lies in its use of fluorescence signal detection, which significantly enhances sensitivity compared with assays that rely on turbidity measurements. In those assays, variations in baseline turbidity among plasma samples can introduce inconsistencies, complicating comparative assessments. In the HoFF test, these variations can be minimized by the standardized manual addition of a fluorescence tracer, ensuring consistency across samples. Notably, the test using infrared fluorophore Alexa647-fibrinogen exhibited a lower background signal from plasma compared with Alexa488-fibrinogen, and can effectively demonstrate fibrinolysis in both plasma clots and whole blood clots. Hence, Alexa647-fibrinogen should be recommended for future studies comparing fibrinolysis in plasma and whole blood.

Additionally, the gradual increase in fluorescence-FDP signal in the HoFF test enables precise mathematical modeling. In contrast, assays based on hemoglobin detection often suffer from scattered and irregular signal outputs due to the sporadic release of red blood cells into the detection area. Furthermore, hemoglobin is not a direct substrate of plasmin, making it more challenging to develop accurate mathematical models for the hemoglobin-based method.

Though fibrinolysis is directly mediated by plasmin, assays that evaluate the generation of plasmin do not necessarily reflect the capacity of the plasmin generated to degrade a fibrin clot, since plasmin generation can also occur in the solution phase. In the presence of fibrin, both tPA and plasminogen dock onto exposed lysine residues, which facilitates the local tPA-mediated plasminogen activation, leading to fibrin degradation. However, when plasminogen is activated in the solution phase, the plasmin generated may not necessarily target fibrin and would also be rapidly sequestered by protease inhibitors (notably α2-antiplasmin). To distinguish fibrinolysis from plasmin generation, we compared the inhibitory effect of TXA on fibrinolysis using the HoFF test and plasmin generation using the S-2251 amidolytic assay. In the presence of TXA, the lysine binding sites on plasminogen were blocked and could not engage fibrin. However, plasminogen in solution was still accessible to be activated by tPA, which led to plasmin formation and cleavage of the soluble plasmin substrate S-2251.

The HoFF test allows for the full extent of clot formation and maturation before thrombolytics are added. Though global clot lysis assay and thromboelastographic assay have been well developed to evaluate thrombolysis and to screen thrombolytic reagents, the advantage of investigating fibrinolysis using established clots in the HoFF test is evident, as it accounts for the critical physical characteristics that emerge from clot maturation. Previous studies suggest the aging of blood clots also contributes to the resistance against thrombolysis, resulting from the cross-linking mediated by factor (F)XIII following thrombus formation [16,17].

In conclusion, we developed a high-throughput, fluorescence-based assay using the degradation of an established fibrin clot as the readout to quantitatively describe the fibrinolytic capacity. This innovative method stands out as a broadly accessible, efficient, and valuable tool for advancing research in fibrinolysis. Given the *ex vivo* nature of this method, the potential for adaptation of this assay into a diagnostic tool is substantial. It also holds promise for personalizing medical care by tailoring treatment strategies based on individual fibrinolytic profiles and for enhancing thrombolytic and antithrombolytic drug screening processes.
